# Preventable trauma deaths in the Western Cape of South Africa: A consensus-based panel review

**DOI:** 10.1371/journal.pgph.0003122

**Published:** 2024-05-10

**Authors:** Julia Dixon, Shaheem de Vries, Chelsie Fleischer, Smitha Bhaumik, Chelsea Dymond, Austin Jones, Madeline Ross, Julia Finn, Heike Geduld, Elmin Steyn, Hendrick Lategan, Lesley Hodsdon, Janette Verster, Suzan Mukonkole, Karlien Doubell, Navneet Baidwan, Nee-Kofi Mould-Millman

**Affiliations:** 1 University of Colorado School of Medicine, Aurora, Colorado, United States of America; 2 Western Cape Government Health and Wellness, Cape Town, South Africa; 3 Colorado Permanente Medical Group, Denver, Colorado, United States of America; 4 Stellenbosch University, Cape Town, South Africa; 5 University of Cape Town, Cape Town, South Africa; 6 Department of Family and Community Medicine, University of Alabama at Birmingham, Birmingham, Alabama, United States of America; Aga Khan University Medical College Pakistan, PAKISTAN

## Abstract

Injury causes 4.4 million deaths worldwide annually. 90% of all injury-related deaths occur in low-and-middle income countries. Findings from expert-led trauma death reviews can inform strategies to reduce trauma deaths. A cohort of trauma decedents was identified from an on-going study in the Western Cape Province of South Africa. For each case, demographics, injury characteristics, time and location of death and postmortem findings were collected. An expert multidisciplinary panel of reviewed each case, determined preventability and made recommendations for improvement. Analysis of preventable and non-preventable cases was performed using Chi-square, Fisher’s exact, and Wilcoxon signed rank tests. A rapid qualitative analysis of recommendations was conducted and descriptively summarized. 138 deaths (48 deceased-on-scene and 90 pre- or in-hospital deaths) were presented to 23 panelists. Overall, 46 (33%) of deaths reviewed were considered preventable or potentially preventable. Of all pre- and in-hospital deaths, late deaths (>24 hours) were more frequently preventable (22, 56%) and due to multi-organ failure and sepsis, compared to early deaths (≤24 hours) with 32 (63%) that were non-preventable and due to central nervous system injury and haemorrhage. 45% of pre and in-hospital deaths were preventable or potentially preventable. The expert panel recommended strengthening community based primary prevention strategies for reducing interpersonal violence alongside health system improvements to facilitate high quality care. For the health system the panel’s key recommendations included improving team-based care, adherence to trauma protocols, timely access to radiology, trauma specialists, operative and critical care.

## Introduction

Injuries are the world’s fifth leading cause of death overall, and the primary cause of death amongst those aged 5 to 29 years [1, 2]. Globally, injuries account for 4.4 million deaths annually, and the World Health Organization (WHO) estimates that 3.1 million injury-related deaths are fully preventable [2].

The burden of injury is disproportionately experienced by young people of low socio-economic status. An estimated 90% of all injury-related deaths occur in low- and middle-income counties (LMICs) [1, 3]. Further, men experience double the risk of injury-related death than women, and the majority of those deaths result from road traffic injuries, homicide, and suicide [1]. Multi-disciplinary expert panel reviews can assess preventability of trauma deaths and identify modifiable and intervenable factors [4, 5]. Yet, such reviews are infrequently performed in most LMICs [5–7].

South Africa is an upper middle-income country with tremendous socio-economic inequalities [8]. Trauma accounts for 10% of all-cause mortality in the nation, with road-traffic injury and homicide mortality rates two and eight times greater than the global average, respectively [9]. The trauma death rate in South Africa of 157.8 per 100,000 is higher than the African continent rate of 139.5 [10]. Public health and subject matter experts have urgently called for action to strengthen emergency care systems to help reduce trauma deaths, especially in the Western Cape which bears a large burden of South Africa’s trauma epidemic [11–14].

*Epidemiology and Outcomes of Prolonged Trauma Care* (*EpiC*) is a prospective multi-centre study of trauma patients in a cross section of the Western Cape public health system [15]. In April 2022, a multi-disciplinary expert panel conducted a comprehensive review of trauma-related deaths identified by the EpiC study to determine rates and factors contributing to preventability. We present key findings and recommendations from this inaugural preventable trauma death review.

## Methods

### Design

We used a multi-methods approach, including an expert panel consensus process to determine preventability and a rapid qualitative analysis of contributory factors and recommendations by the experts. Preventability was determined using a categorization scheme compiled from other peer-reviewed publications, and WHO guidelines (Table 1) [4, 16, 17].

**Table 1:** Definitions of preventability determinations [4, 15, 16].

Determination	Description
Preventable (or definitely survivable)	Minimal anatomic injuries with a high likelihood of survival. Injuries and sequelae considered to be survivable: Death could have been prevented if appropriate steps had been taken; Frank deviations from standard of care that directly or indirectly caused death; Statistical probability of survival greater than 50% [4, 15, 16].
Potentially preventable (or potentially Survivable)	Anatomic injuries that were severe but medically survivable injuries and sequelae severe but survivable: Death potentially could have been prevented if appropriate steps had been taken; Evaluation and management generally appropriate; Some deviations from standard of care that may directly or indirectly have been implicated in death; Statistical probability of survival 25–50% [4, 15, 16].
Non-preventable (or non-survivable)	Death as a result of catastrophic anatomic injuries: Injuries and sequelae non-survivable even with optimal management; Evaluation and management appropriate according to accepted standards; If patient had co-morbid factors, these were major contributions to death; Statistical probability of survival less than 25% [4, 15, 16].
Non-preventable (but with care that could have been improved)	As with non-preventable above, but care is questionable or clear errors in care are detected, even though these did not lead to death [4,15,16].
Indeterminate	Information insufficient to make a determination [4].

### Study population and setting

The Western Cape Province is located on the southwestern corner of South Africa with an estimated population of 7.1 million and endure high rates of physical trauma, interpersonal violence, unemployment and poverty [12, 18–21]. EpiC study sites are located in two municipalities: City of Cape Town Metropolitan Municipality and Cape Winelands District Municipality. The metropolitan district is a dense urban area that houses the Cape Town city center, inclusive of informal settlements. The total population is estimated at approximately 3.7 million [18]. The metro area study sites include Tygerberg Hospital (trauma referral center), Khayelitsha (District) Hospital, Western Cape Government Emergency Medical Services (WCG EMS) bases, and Tygerberg Forensic Pathology Service (FPS) laboratory. The Cape Winelands District is characterized by a mixture of urban, peri-urban, and rural municipalities that receive care at Ceres (District) Hospital, Worcester Regional Hospital, two WCG EMS bases, and Worcester FPS Laboratory. The Cape Winelands District includes several smaller municipalities with populations averaging 158,000 each [18]. In South Africa, all unnatural deaths, inclusive of traumatic deaths, are required to undergo autopsy per the Inquests Act (R.359 of the National Health Act, 2018).

### Case inclusion/exclusion

All trauma deaths captured by the EpiC study from 1 January– 31 December 31, 2021 were included in the expert panel review, in addition to 60 randomly selected deaths that were declared deceased-on-scene within the EpiC study catchment and made available by the EpiC study pathologist co-Investigators. Deceased-on-scene cases were included to ensure representation of deaths that occurred prior to any contact with the health care system. Cases excluded in the EpiC study, and therefore excluded from this review, were less than 18 years of age, prisoner, injury occurred >24-hours prior to initial medical encounter, envenomations, toxicologic injuries, drownings, seen at a non-study site, and/or transported via private EMS agency [15].

### Expert panel and review process

The panel comprised 23 multidisciplinary subject matter experts familiar with the local emergency and healthcare system. Panelists included: 4 trauma surgeons, 1 neurosurgeon, 2 intensive care and burns specialists, 4 forensic pathologists, 4 prehospital care experts, 6 emergency medicine specialists, and 2 family medicine specialists. Case summaries were prepared using all available clinical documents including records from EMS, hospital units, surgical theatre, laboratory and radiology, and post-mortems. For logistic feasibility and efficiency, panelists were divided into two even groups with balanced subject-matter expertise for case presentations and discussions. Consensus on death preventability was achieved if 80% of the panel agreed by an open show-of-hands vote. Any cause of death that was categorized as catastrophic tissue destruction was automatically categorized as non-preventable, consistent with prior preventable trauma death panel reports [7, 17, 22–24].

### Data collection

Patient demographics, injury details, time and location of death, and postmortem findings were recorded from health records. For deceased-on-scene cases, only post-mortem reports and police reports were available for review, hence only demographics, injury force type, preventability and physiologic cause of death were recorded.

The major categories for physiologic cause of death were catastrophic tissue destruction (CTD), central nervous system (CNS), haemorrhage, multi organ failure and/or sepsis (MOF+S), comorbidities and ‘other’ (Table 2) [15, 23, 25, 26]. This classification system was adapted from prior mortality reviews and modified by an expert panel Delphi process for this panel [15, 23, 26]. During the review panel, data collectors recorded the consensus panel determination of preventability, physiologic cause of death, and panellist recommendations.

**Table 2:** Physiologic cause of death [15, 23, 25, 26].

Major category	Sub-categories
Catastrophic tissue destruction (CTD)	Total body (physical dismemberment) Brain Cardiac Open pelvis Extremity amputation Abdominal aorta Thoracic aorta Incineration Other (major vessel, liver, trachea)
Central nervous system (CNS) injury	Brain Brain stem High cervical spine (at or higher than C3)
Hemorrhage or exsanguination	Truncal Extremity Junctional
Multiple organ failure + Sepsis (MOF+S)	Brain Cardiac failure Coagulopathy Liver failure Pulmonary failure Renal failure Sepsis
Comorbidities	• A significant underlying (medical) disease that directly caused death
‘Other’	Airway Breathing Lung (i.e., penetrating lung injury impairing airway & breathing with hemorrhage) Cardiac tamponade Tension pneumothorax Pulmonary embolism Full thickness burns/incineration Physiologic collapse Sequalae of injury Other (including iatrogenesis)
Unknown	• No cause identified

### Quantitative analysis

Demographic and injury characteristics were analysed descriptively. Individual injuries were coded by the EpiC study using the abbreviated injury scale (AIS), up to a maximum of 10 codes. Each injury was either associated or not associated with the physiologic cause of death. We performed Chi-square and Fisher’s exact tests to compare preventable and non-preventable deaths for the entire cohort. Fisher’s exact tests were used when more than 20% of expected values were less than 5. A p-value ≤0.05 determined significance. Following a globally significant p-value, chi-square tests were performed for specific two-group comparisons. All analyses were conducted using SAS Software (version 9.4, SAS Institute Inc). We provided estimates and corresponding 95% Confidence Intervals from unadjusted generalized linear models analyzing the associations between covariates of interest and preventability (Preventable vs Non-Preventable) as the outcome. For the comparative analysis, the categories of preventable and potentially preventable were combined, and non-preventable with or without improved care and indeterminate were combined.

**Fig 1:**
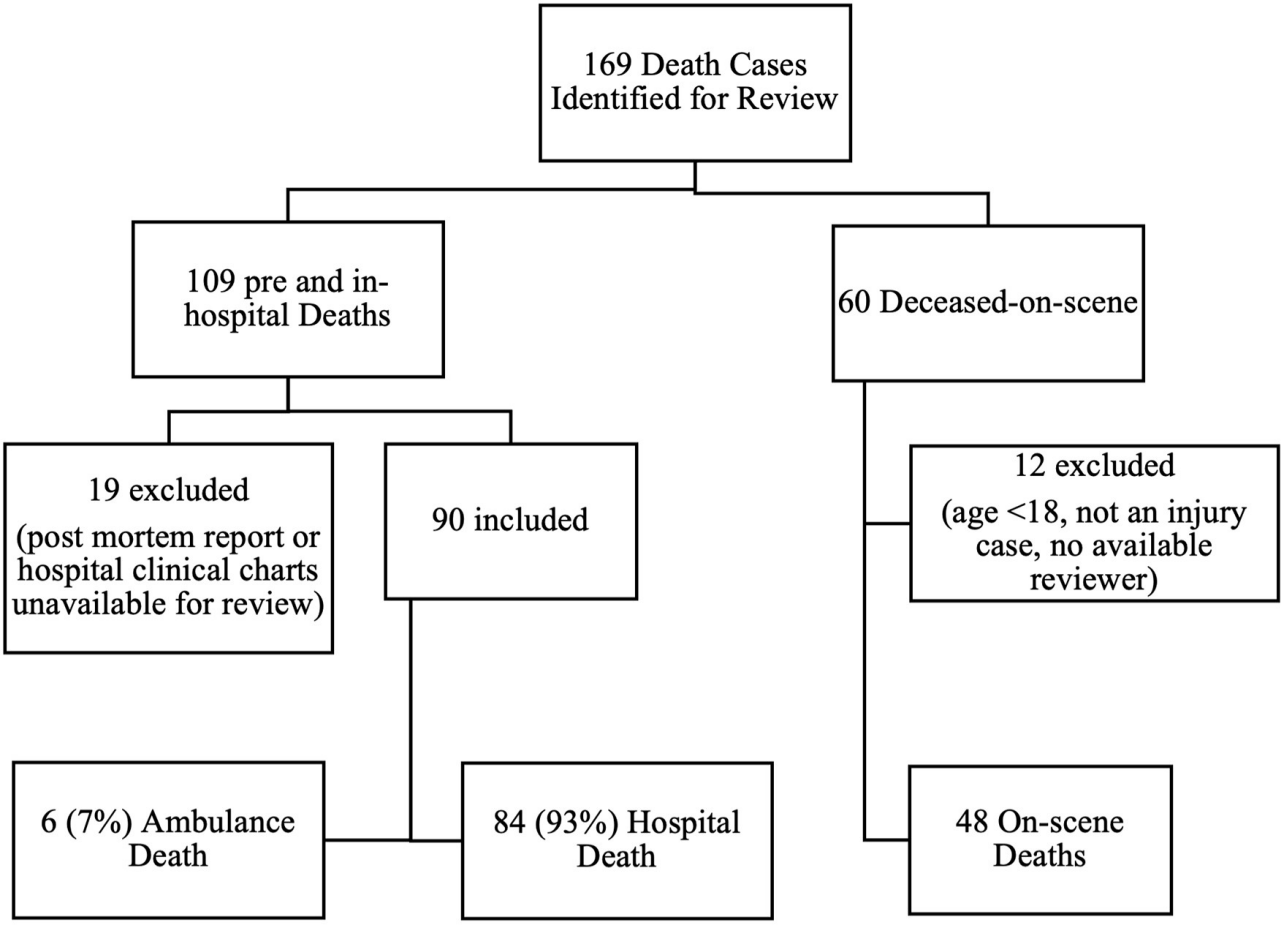
Enrolment flowchart.

169 decedents were identified for initial review (109 pre- and in-hospital and 60 deceased-on-scene). A total of 138 cases were reviewed by the panel (Fig 1).

### Qualitative analysis

A rapid qualitative assessment of opportunities for improvement was performed [27]. Notes on the expert panel’s identified opportunities for improvement and recommendations were analyzed by two co-investigators (REDACTED). Thematic codes were created and assigned to each recommendation and then categorized and mapped into a matrix organized according to the applicable level of the health system and the descriptive category for improvement and assigned to each comment (S1 Appendix). Codes were also descriptively summarized by categories including injury characteristics, preventability and health system levels.

### Ethics

All study activities were pre-approved by the Health Research Ethics Committee at Stellenbosch University (Ethics Reference No. N20/03/036), the individual health facilities and agencies, and the Western Cape Provincial Health Research Committee. A waiver of informed consent was used for this study as approved by Stellenbosch University Human Research Ethics Committee (SUHREC) with a reliance agreement provided by the Colorado Multiple Institute Review Board (COMIRB #20–2176). As approved by SUHREC, study team members retrospectively reviewed patient hospital and decedent records which include patient identifying information. For this study records were accessed between 1 January 2021 and 30 April 2022.

## Results

The median age was 32 years and the majority were men (83.3%) (Table 3). Penetrating injuries were the dominant injury force type (47%), followed by blunt force injuries (39%). Firearms were the leading mechanism of injury representing 33% of deaths. Other leading mechanisms were vehicular, including pedestrian versus auto (23%); interpersonal penetrating trauma, including stabs (20%); and interpersonal blunt force trauma, including community assault (16%). Of the 90 pre- and in-hospital deaths reviewed 58 (64%) occurred at the tertiary hospital, 20 (22%) at a district hospital, 6 (7%) at a regional hospital and 6 (7%) in an ambulance. The largest proportion of preventable deaths occurred at the tertiary hospital (67%) followed by district hospitals (11%). Overall, there was no significant difference in demographics, injury force type, or mechanism between the preventable and non-preventable death groups (Table 3).

**Table 3:** Characteristics of non-preventable versus preventable deaths.

Characteristic	Total n = 138 N(%)	Non-preventable^#^ and indeterminate n = 92 N (%)	Preventable and potentially preventable^@^ n = 46 N (%)	P-value
Median Age (IQR)	32 (27.4–37.7)	31.0 (27.2–37.0)	33.9 (28.6–44.5)	0.08
Sex				
*Male*	115 (83.3)	74 (80.4)	41 (89.1)	0.20
*Female*	23 (16.7)	18 (19.6)	5 (10.9)	
**Injury force type**				
*Blunt*	54 (39.1)	34 (37.0)	20 (43.5)	0.90
*Penetrating*	65 (47.1)	45 (48.9)	20 (43.5)	
Mixed*	10 (7.2)	7 (7.6)	3 (6.5)	
Others**	9 (6.5)	6 (6.5)	3 (6.5)	
**Injury mechanism**				
*Firearm*	46 (33.3)	35 (38.0)	11 (23.9)	0.42
*Stab/cut*	32 (23.2)	16 (17.4)	11 (23.9)	
*Struck/hit*	22 (15.9)	13 (14.1)	9 (19.6)	
*Vehicular injury*	27 (19.6)	22 (23.9)	10 (21.7)	
*Others*	11 (8.0)	6 (6.5)	5 (10.9)	
**Physiologic Cause of Death**				n/a
*CTD*	46 (33.3)	46 (50.0)	n/a	
*CNS*	23 (16.7)	19 (20.7)	4 (8.7)	
*Hemorrhage*	28 (20.3)	13 (14.1)	15 (32.6)	
*MOF+S*	19 (13.8)	2 (2.2)	17 (37.0)	
*Other & Comorbidities*	22 (15.9)	12 (13.0)	10 (21.7)	
***Location of Death***				n/a
*Tertiary Hospital*	58 (42.0)	27 (29.3)	31 (67.4)	
*Regional Hospital*	6 (4.3)	3 (3.3)	3 (6.5)	
*District Hospital*	20 (14.5)	15 (16.3)	5 (10.9)	
*EMS*	6 (4.3)	4 (4.3)	2 (4.3)	
*On Scene*	48 (34.8)	43 (46.7)	5 (10.9)	

IQR = Interquartile Range, CTD = catastrophic tissue destruction, CNS = central nervous system, MOF+S = multi organ failure and sepsis.

*Mixed included cases with both blunt and penetrating injuries.

**Other includes burns, traumatic amputations.

^#^The non-preventable group includes indeterminate cases.

^@^The preventable group includes potentially preventable cases.

### Preventability

12 (9%) deaths were classified as preventable and an additional 34 (24%) as potentially preventable (Table 4). For preventable and potentially preventable cases, the panel discussed the importance of a team-based approach to trauma care, including collaboration among pre-hospital providers, physicians, and nurses to facilitate early recognition of critical illness and appropriate initial resuscitation. Delayed access to prehospital services, advanced radiology, specialty and operative care also contributed to preventable deaths.

**Table 4:** Demographic characteristics, stratified by preventability, for 138 trauma-related deaths in the Western Cape, South Africa.

Characteristic	Indeterminate N (%)	Non-preventable N (%)	Non-preventable (with improvements) N (%)	Potentially preventable N (%)	Preventable N (%)	Overall N (%)
Age [IQR]	[23.0–35.0]	[28.0–37.3]	[24.9–34.3]	[27.3–44.5]	[29.2–43.1]	[27.4–37.7]
Sex						
*Male*	3 (100.0)	49 (77.8)	22 (84.6)	30 (88.2)	11 (91.7)	115 (83.3)
*Female*	0 (0.0)	14 (22.2)	4 (15.4)	4 (11.8)	1 (8.3)	23 (16.7)
Region						
*Cape Winelands*	0	13 (20.6)	2 (7.7)	6 (17.7)	1 (8.3)	22 (16.0)
*City of Cape Town*	4 (100)	50 (79.4)	24 (92.3)	28 (82.4)	11 (91.7)	116 (84.1)
Injury force type						
*Blunt*	1 (25.0)	19 (30.2)	14 (53.8)	13 (38.2)	7 (58.3)	54 (39.1)
*Penetrating*	0 (0.0)	35 (55.6)	10 (38.5)	15 (44.1)	5 (41.7)	65 (47.1)
*Blunt & Penetrating*	2 (66.7)	5 (7.9)	0 (0.0)	3 (8.8)	0 (0.0)	10 (7.2)
*Others*	0 (0.0)	4 (6.3)	2 (7.7)	3 (8.8)	0 (0.0)	9 (6.5)
Injury mechanism						
*Firearm*	0 (0.0)	28 (44.4)	7 (26.9)	11 (32.3)	0 (0.0)	46 (33.3)
*Stab/cut*	1 (25.0)	12 (19.0)	3 (11.5)	6 (17.6)	5 (41.7)	27 (19.6)
*Struck/hit*	2 (50.0)	6 (9.5)	5 (19.2)	5 (14.7)	4 (33.3)	22 (15.9)
*Vehicular injury*	0 (0.0)	15 (23.8)	7 (26.9)	7 (20.6)	3 (25.0)	32 (23.2)
*Others*	0 (0.0)	2 (3.2)	4 (15.4)	5 (14.7)	0 (0.0)	11 (8.0)
**Total**	3 (2.0)	63 (46.0)	26 (19.0)	34 (24.0)	12 (9.0)	138 (100.0)

Overall, 4 (1%) deaths had an indeterminate preventability due to a lack of documented clinical details in the records. Only one death was directly attributed to comorbidities, specifically chronic obstructive pulmonary disease, and was classified as a preventable death and analysed as part of the other mechanisms of death category.

### Physiologic cause of death

**Fig 2:**
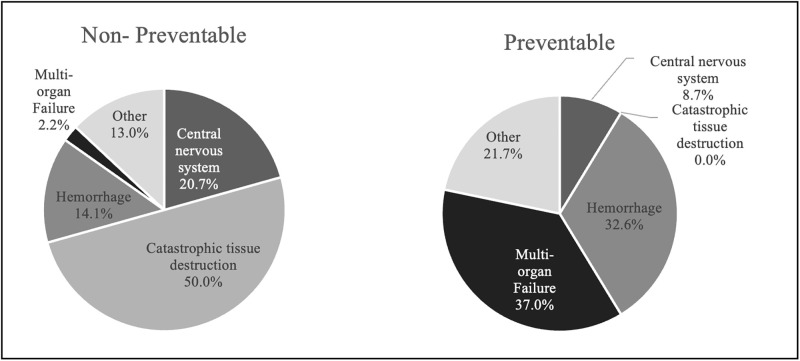
Physiologic cause of death and preventability.

Among all deaths, the leading physiologic cause of death was CTD (33%). Among preventable deaths, the most common physiologic cause of death was MOF+S (17, 36.2%) and for non-preventable deaths, the majority (46, 50%) were CTD (Fig 2). The majority of CTD deaths were from brain injuries (16, 70%) and brain stem injuries (5, 22%) (S2 Appendix). Amongst haemorrhage deaths, the most frequent sub-categories were truncal (18, 44%) and junctional (9, 32%). For those who survived to ambulance or hospital care, the leading physiologic cause of death was evenly distributed across three major categories: CNS (19, 21%), haemorrhage (19, 21%), and MOF+S (19, 21%). For those who died on scene, the leading physiologic causes of death were catastrophic tissue destruction (30, 63%) and haemorrhage (9, 19%).

For pre- and in-hospital deaths, the panel identified poor recognition of critical vital signs and delays in care as contributors to poor outcomes, which should be addressed through implementation and adherence to clinical protocols, increased patient monitoring, and utilization of a team-based approach to trauma care. For CTD deaths, the expert panel agreed the main contributing factors were alcohol, interpersonal violence and a lack of road safety highlighting the need for primary prevention strategies at the community level.

### Timing of deaths

**Fig 3:**
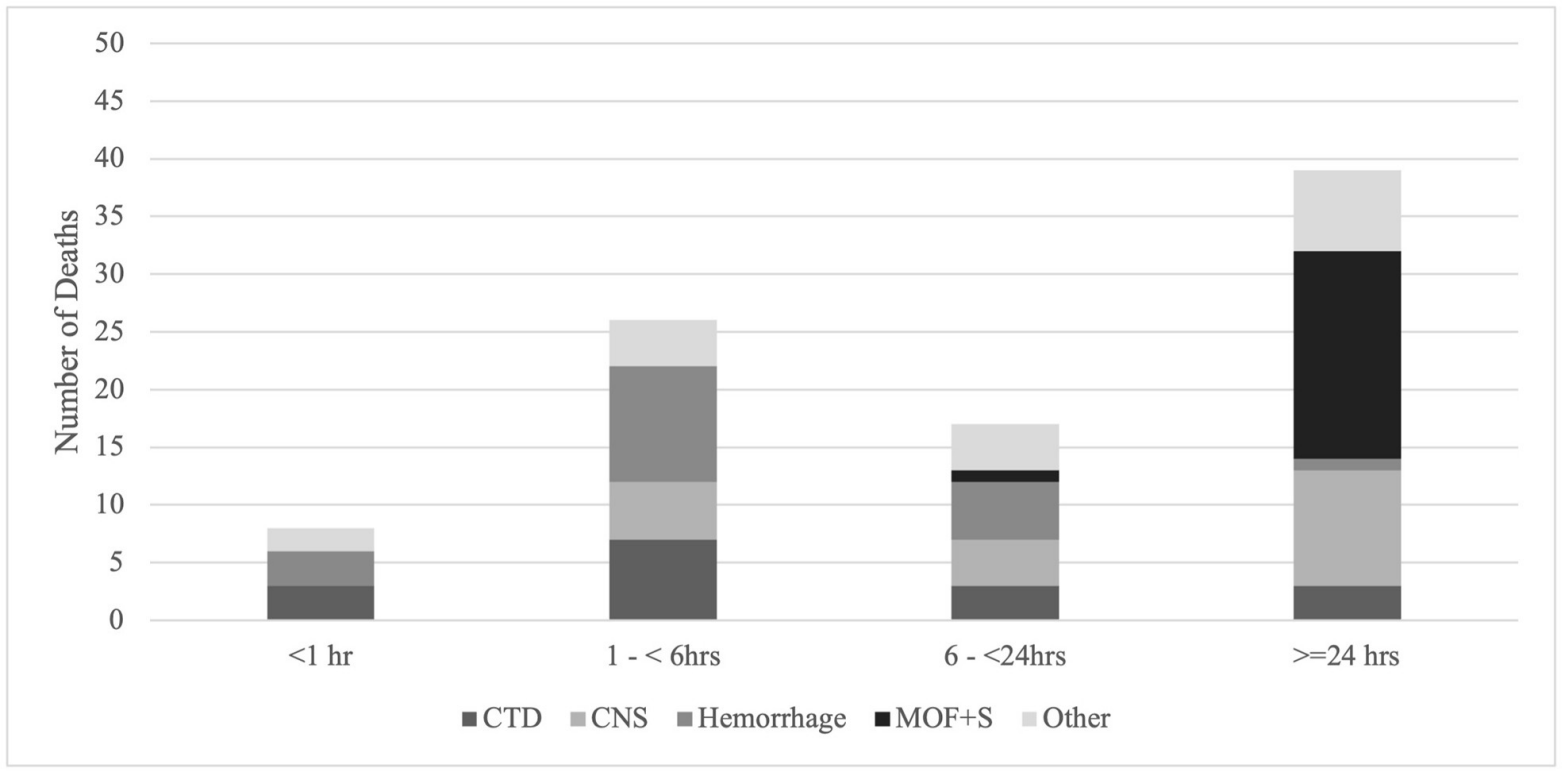
Hours to death and physiologic cause of death.

**Fig 4:**
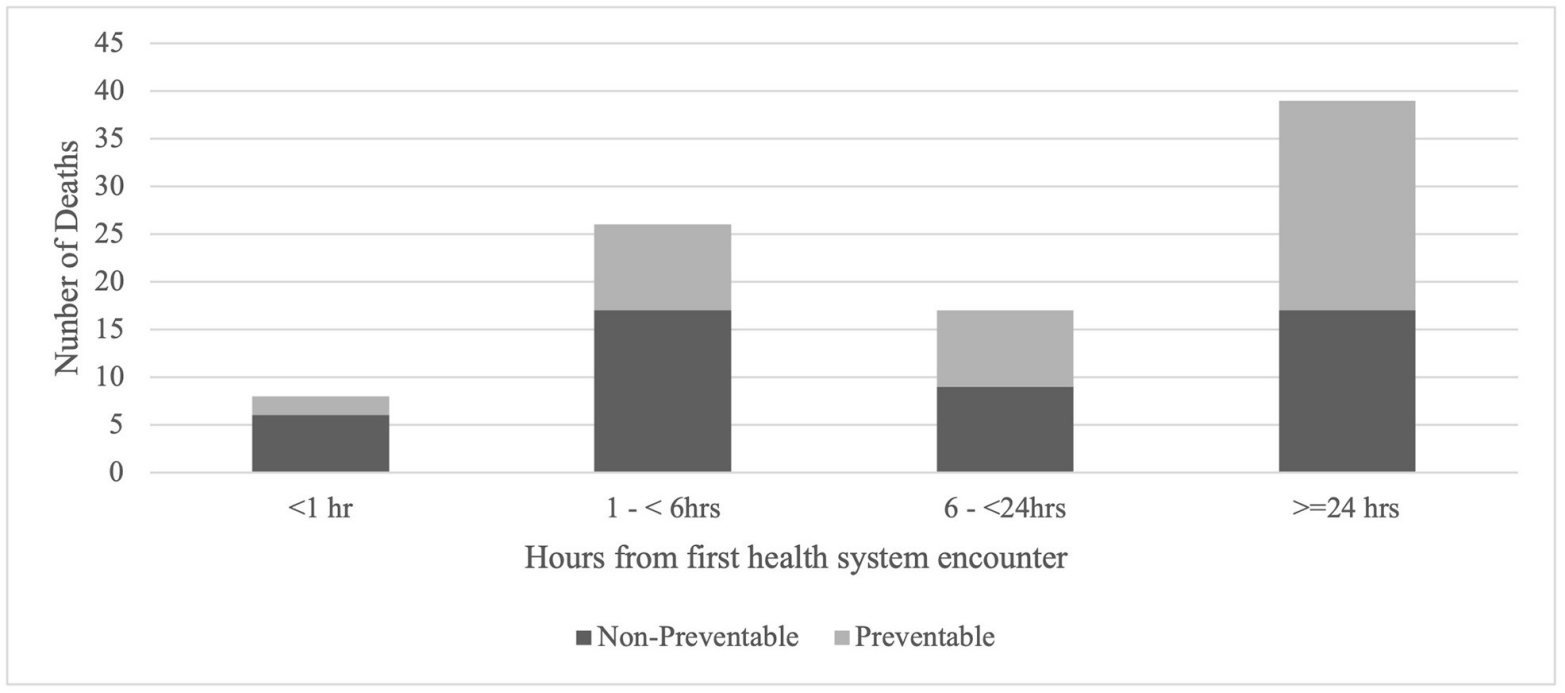
Hours to death and preventability.

Among pre- and in-hospital deaths, the median time from first encounter with a health care provider (either EMS or hospital) to death was 16.8 hours (IQR 2.9–91.9 hours). The median time to death for non-preventable deaths was 9.5 hours (IQR 2.4–44.1) and for preventable deaths 29.5 hours (IQR 3.8–216.1). Among pre- and in-hospital deaths, there were two peaks of death: at 1–6 hours and >24-hours (Fig 3). Early deaths (<6 hours) were most often due to catastrophic CNS injuries (26%) and truncal haemorrhage (26%). Late deaths (>24 hours) were most often due to MOF+S (46%) and CNS-brain (21%). 54% of the deaths occurring after 24 hours were considered preventable compared to 27% of deaths that occurred in less than 6 hours (Fig 4). Improved provider knowledge and recognition of critical diagnoses was recommended for the early preventable deaths in conjunction with increased staffing of nurses and both junior and senior providers. For the late preventable deaths, strategies to optimize early recognition and management of infectious complications, and most importantly, reduction of in-hospital time waiting for definitive surgery are needed. Deceased-on-scene cases were not included in this analysis due to uncertainty surrounding the exact time of injury.

### Injury body region

Among pre- and in-hospital deaths individual injuries were assigned an AIS. The most frequently injured body regions, as classified by AIS injury body regions, were the head and face (250, 38.6%) and the thorax and abdomen (238, 36.8%). In haemorrhage deaths, the most frequently injured body region was the thorax and abdomen (63, 40.4%) (Table 5). For deaths due to haemorrhage, the factors most frequently discussed for improvement were lack of access to radiology, specialist and operative care at the district and regional level and timelier access at the tertiary level. At the provider and hospital unit level the panel identified poor or delayed recognition of critical illness and gaps in early resuscitation as frequently contributing factors.

**Table 5:** a and b: Frequencies of AIS body regions.

**A**						
**Frequencies of AIS body region injuries contributing to cause of death**						
**Physiologic cause of death (PCOD)**	**Body Regions Injured (associated with PCOD)**					
	**Head & Face**	**Neck**	**Thorax & Abdomen**	**Upper & Lower Extremity**	**Spine**	**External and Other**
**CTD**	28	0	24	3	0	7
**CNS**	5	3	12	2	0	3
**Hemorrhage**	11	2	23	0	2	6
**MOF**	13	0	7	0	0	2
**Other**	12	0	9	0	11	0
**Total**	**69**	**5**	**75**	**6**	**13**	**18**
**B**						
**Injury Frequencies by AIS Body Region**						
**Physiologic cause of death (PCOD)**	**Body Regions Injured (associated with PCOD)**					
	**Head & Face**	**Neck**	**Thorax & Abdomen**	**Upper & Lower Extremity**	**Spine**	**External and Other**
**CTD**	106	1	80	25	6	15
**CNS**	20	8	37	13	1	6
**Hemorrhage**	44	6	63	20	11	12
**MOF**	50	2	24	6	1	5
**Other**	30	0	34	13	2	6
**Total**	**250**	**17**	**238**	**77**	**21**	**44**

CTD = catastrophic tissue destruction, CNS = central nervous system, H = haemorrhage, MOF+S = multi organ failure and sepsis.

### Factors associated with preventability

Findings from univariate models analyzing the association between characteristics of interest and preventability are found in Table 6. There was no significant odds of preventability related to injury mechanism or injury force. The odds of having preventable death were 82% lower among those that were due to CNS vs hemorrhage (OR 0.18, CI 0.05–0.68), 98% lower among CNS vs MOF+S (OR 0.02, CI 0.00–0.15) (Table 5). The odds of preventable death from MOF+S were over 7 times higher when compared to hemorrhage (OR 7.37 CI 1.42, 38.08). Deaths that occurred less than 6 hours from injury were more likely to be non-preventable (OR 0.20, CI 0.08, 0.52). Recommendations from the panel for non-preventable deaths were most frequently related to trauma prevention and the need for strategies to reduce inter-personal violence. For preventable deaths from sepsis, the panel recommended increased nursing and physician staffing in both emergency and inpatient units as well as improving resources at hospital unit level to ensure early and effective surgical management of bleeding and contamination to reduce the risk of development of MOF+S.

**Table 6:** Univariate models analyzing the association between characteristics of interest and preventability (preventable vs non-preventable).

Characteristic	Odds Ratio & 95% Confidence Interval*	p-value
Age categorized		
≤ 32 years vs > 32 years	0.56 (0.28, 1.16)	0.12
Sex		
Female vs Male	1.99 (0.69, 5.77)	0.20
Injury mechanism		
Firearm vs Vehicular	0.69 (0.25, 1.90)	0.42
Stab/cut vs Vehicular	1.51 (0.52, 4.42)	
Struck/hit vs Vehicular	1.52 (0.49, 4.72)	
Other vs Vehicular	1.83 (0.45, 7.45)	
Injury force		
Blunt vs Penetrating	1.32 (0.62, 2.84)	0.47
Physiologic cause of death		
CNS vs Hemorrhage	0.18 (0.05, 0.68)	**0.01**
CNS vs MOF	0.02 (0.00, 0.15)	**<0.001**
MOF vs Hemorrhage	7.37 (1.42, 38.08)	**0.02**
Timing of Death		
<6 hours vs >24 hours	0.21 (0.09, 0.49)	**<0.001**

NISS = New Injury Severity Score, CNS = Central Nervous System, MOF = Multiple Organ Failure.

*An odds ratio (OR) <1 represents an increased likelihood of non-preventability in the primary category and OR >1 trending towards preventable in the primary category.

## Discussion

In this study, an expert panel of local clinicians evaluated the preventability of injury-related deaths in the Western Cape Province of South Africa. Of the pre- or in-hospital trauma deaths, 45% were categorized as preventable or potentially preventable, and deaths were mostly consequent to interpersonal violence, firearms and vehicular injuries. Among preventable deaths, the majority occurred at the tertiary hospital and the most frequent physiologic causes of death were MOF+S and haemorrhage, both truncal and junctional. Injuries to the brain and brain stem accounted for a large portion of non-preventable deaths. Panellists’ key recommendations were quality team-based care and timely access to (1) prehospital care, (2) emergency radiology and (3) operating theatre to manage haemorrhage and contamination. This is consistent with prior work that highlights the need for access to prehospital services, diagnostics and definitive care in order to address the high rate of preventable mortality in low resource settings [28].

Inter-personal violence involving firearms or physical assault was a dominant factor in the majority of deaths reviewed. Yet, injury prevention requires consideration of a broad set of influences such as poverty and gang culture, violence, alcohol or substance abuse, governance of firearms, protections from intimate partner violence and road safety [29]. South African studies have also shown that the burden of interpersonal violence largely resides with men, during weekends, and is strongly associated with alcohol and substance use [19, 30]. There is a dire need for strengthening community-based primary preventive interventions to decrease the burden of trauma consequent to inter-personal violence and alcohol abuse [8, 20, 21].

MOF+S (37%) was the most frequent physiologic cause of death in preventable cases, with the majority being directly related to sepsis as a complication of traumatic injury. Severe injury, through a broad range of factors and mechanisms, is often complicated by MOF which subsequently increases the risk of infections [31]. Trauma patients with sepsis exhibit reported mortality rates of 20–30%, increased ICU admissions and longer lengths of stay, and increased utilization of health system resources [32, 33]. One trauma death review in Ghana noted a decrease from 6% to 1% in trauma deaths from sepsis after an initial death review resulted in an educational intervention and implementation of sepsis protocols [34]. In addition to decreasing deaths, preventing MOF and sepsis will decrease morbidity and the burden on the limited resources in health systems in LMICs [28, 31, 33]. In this south African setting, the tertiary hospital receives patients requiring specialty care, such as neurosurgery, and/or admission to the ICU. This is one possible explanation for why the majority of preventable deaths occurred at the tertiary hospital in this study.

The second most common cause of preventable death was haemorrhage, with the most frequent body regions being the thorax and abdomen. The primary goal of traumatic haemorrhage management is to stop the bleeding and resuscitate with blood products [35]. Bleeding injuries to the torso are a frequent cause of preventable mortality due to the non-compressible location and the need for rapid definitive management in the operating theatre. Similar to reports from other LMICs, this operative care was often not urgently available in the Western Cape [36]. It is likely that improved access to radiology and theatre, specifically at the district level can help reduce haemorrhage deaths [37–40].

Deaths from brain and brain stem injuries were largely determined to be non-preventable which is consistent with other trauma panel reviews [41, 42]. Even though these deaths were deemed mostly non-preventable, they were noted to add significantly to the local burden of disease and consume substantive health care system resources, including EMS for interfacility transports, radiology, and critical care beds. While computerized tomography (CT) scans remain standard of care, novel low-resource imaging techniques and biomarkers could aid in earlier decision making [43–45]. Early identification of patients unlikely to benefit from further resource consumption is a necessary consideration.

Among those who survive to receive health care, there were two time peaks of death, the first occurring less than 6 hours and the second after 24 hours. Previous studies have linked early deaths to critical and catastrophic injuries that are non-preventable with a smaller number of cases being potential preventable with improved access to high quality care [46, 47]. Higher rates of preventability for delayed deaths are more commonly from MOF+S that may be prevented by improved resuscitation, early surgery and critical care [46, 47]. Thus, our physiologic cause of death findings, as well as the corresponding rates of preventability of 18% among early deaths and 63% among late deaths, appear to correlate.

## Conclusion

In this South African population, an expert panel determined that 45% of pre- and in-hospital deaths were preventable. Early deaths were more frequently due to CNS injuries and haemorrhage. Later deaths were most often due to MOF+S. Experts identified several key areas for improvement including a major focus on primary prevention strategies at the community level relating to substance use and interpersonal violence. Additionally, experts identified several opportunities for strategic improvement, including implementation of, and adherence to, trauma clinical protocols at all facilities, fostering and facilitating a team-based approach to trauma care, and improved access to radiology, surgical and specialty care. Our findings elucidate gaps in clinical care and provide actionable recommendations to improve care and reduce post-injury mortality.

## Limitations

This review assessed only a subset of trauma deaths that occurred in the Western Cape during the study time period and in health systems identified by the EpiC study. Published works from the Western Cape South Africa noted lower trauma burdens during COVID-19 lock downs but rapid returns to pre-pandemic trauma volumes. There was limited data available for deaths that occurred on scene given they had no contact with the health system, and, therefore deceased on scene cases were excluded from the more detailed physiologic analysis.

## Supporting information

S1 Appendix Qualitative coding matrix for panel identified areas of improvement and recommendations. (DOCX)

S2 Appendix Physiologic cause of death for non-preventable versus preventable deaths. (DOCX)

S1 Checklist (DOCX)
