# Supplementary material for: Preventable trauma deaths in the Western Cape of South Africa: A consensus-based panel review
Source: PLOS Glob Public Health. 2024 May 10;4(5):e0003122. doi: 10.1371/journal.pgph.0003122 (PMC11086906; doi:10.1371/journal.pgph.0003122)
Supplement: S1 Appendix — (DOCX) [file pgph.0003122.s001.docx]

**S1 Appendix: Qualitative Coding Matrix for panel identified areas of improvement and recommendations**

| **Level of Deficiency (within health system):** | **Communication & Documentation** | **Clinical Care** | **Resources** | **Policy, Guidelines, or Protocols** |
| --- | --- | --- | --- | --- |
| **Health System** | - Communication between EMS and facilities. - Standard referral processes/channels to facilitate communication. - Documentation of injury incident time. |  | - Care impacted by lack of access to radiology (CT scanners; radiographers; radiologists). - Care impacted by lack of access to theatre. - Care impacted by lack of access to critical care beds - Care impacted by lack of access to trauma care beds. - Limited EMS availability (ALS, IFTs, rural etc). - Need for hemodialysis units/resources. | - Lack of central trauma bed management protocols. - Absent or poor inter-facility trauma referral pathways - Absent or unused EMS trauma referral protocols - Absent system-wide Crush protocol. - Protocols for penetrating head injuries. |
| **Facility/**  **Organization** |  |  | - Access to OT at district level. - Access to OT at tertiary level. - Access to radiology at district level. - Access to radiology at tertiary level. - Lack of critical care beds at select district level. - Access to critical care beds at tertiary level. | - EMS ALS dispatch criteria. - EMS ‘load and go’ policies. |
| **Unit(s)** | - Handover | - Lack of clinical providers/no ownership of admitted patients. - Culture of poor adherence to clinical protocols. | - Access to timely specialty care. - Access to critical care beds. - Access to patient monitoring. - Access to medications/blood products. | - Absent palliative care/de-escalation of care protocols. - Poor team-based trauma care. - No Crush treatment protocols. - No massive blood transfusion guidelines or protocols. - No hypothermia management protocols. - SOPs for nursing monitoring and documentation |
| **People (providers)** | - Missing/poor provider documentation. - Inattention to vital signs. | - Missed or delay in diagnosis (not Crush) - Missed or delay in diagnosis (Crush Syndrome) - General quality of care concern e.g., re-warming, anchoring biases, poor ATLS. - Prolonged EMS scene times. - Gaps in early resuscitation: - Basic resuscitation - Antibiotics | - Inadequate number of burn specialists. - Inadequate number of spinal cord injury specialists. - Inadequate number of intensivists. - Inadequate number of surgery inpatient providers. - Inadequate nursing (high ratios, low numbers in units, etc) | - Lack of awareness or adherence to guidelines. |
| **Deficiency level (outside the health system):** | **Communication & Documentation** | **Education** | **Community Factors** | **Policies** |
| **System (transport, police, legal, etc)** | - Missing or poor police/scene documentation. |  | - Community assault as method of self-policing. | - Gun legislation/gun control. - Road Safety, primary prevention (PVA). - Inadequate ‘Red Zone’ policing/security. |
| **People**  **(community)** |  | - Alcohol abuse. - Interpersonal violence. - Road Safety (esp. PVA). - Gun Safety. - Decision to seek care. - Decisions to call EMS. | - Socioeconomics of interpersonal violence. - Gender based violence. - Barriers to accessing care. - Lack of community mental health resources. | - Alcohol abuse in community. - Interpersonal violence (IPV): - Firearms - Knife/stabs - Community Assault - Poor pedestrian road safety culture/poor awareness on PVAs. |
