# Supplementary material for: Preventable trauma deaths in the Western Cape of South Africa: A consensus-based panel review
Source: PLOS Glob Public Health. 2024 May 10;4(5):e0003122. doi: 10.1371/journal.pgph.0003122 (PMC11086906; doi:10.1371/journal.pgph.0003122)
Supplement: S2 Appendix — (DOCX) [file pgph.0003122.s002.docx]

**S2 Appendix:** Physiologic cause of death for non-preventable versus preventable deaths

| **Physiologic Cause of Death** | | **Total n=138**  **N(%)** | | **Non-preventable^#^ and indeterminate n=92 N (%)** | | **Preventable and potentially preventable^@^ n=46**  **N (%)** | | |
| --- | --- | --- | --- | --- | --- | --- | --- | --- |
| *CTD-Total Body* | | 6 (4) | | 6 (13) | | n/a |  |  |
| *CTD-Brain* | | 25 (18) | | 25 (54) | | n/a |  |  |
| *CTD-Brain Stem* | | 1 (1) | | 1 (2) | | n/a |  |  |
| *CTD-Cardiac* | | 8 (6) | | 8 (17) | | n/a |  |  |
| *CTD-Incineration* | | 2 (1) | | 2 (4) | | n/a |  |  |
| *CTD-Thoracic Aorta* | | 3 (2) | | 3 (7) | | n/a |  |  |
| *CTD-Other* | | 1 (1) | | 1 (2) | | n/a |  |  |
| *CNS-Brain* | | 16 (12) | | 13 (28) | | 3 (3) |  |  |
| *CNS-Brain Stem* | | 5 (4) | | 4 (9) | | 1 (1) |  |  |
| *CNS-High Cervical Spine* | | 2 (1) | | 2 (4) | | 0 |  |  |
| *H-Truncal* | | 18 (13) | | 7 (15) | | 11 (12) |  |  |
| *H-Junctional* | | 9 (7) | | 5 (11) | | 4 (4) |  |  |
| *H-Extremity* | | 0 | | 0 (0) | | 0 |  |  |
| *H-CA* | | 1 (1) | | 1 (2) | | 0 |  |  |
| *MOF-Coagulopathy* | | 1 (1) | | 0 | | 1 (1) |  |  |
| *MOF-Pulmonary* | | 1 (1) | | 0 | | 1 (1) |  |  |
| *MOF-Renal* | | 3 (2) | | 0 | | 3 (3) |  |  |
| *MOF-Sepsis* | | 14 (10) | | 2 (4) | | 12 (13) |  |  |
| *C-COPD* | | 1 (1) | | 0 | | 1 (1) |  |  |
| *O-Airway* | | 3 (2) | | 3 (7) | | 0 |  |  |
| *O-Cardiac Tamponade* | | 1 (1) | | 1 (2) | | 0 |  |  |
| *O-Tension Pneumothorax* | | 5 (4) | | 0 | | 5 (5) |  |  |
| *O-Pulmonary Embolism* | | 1 (1) | | 0 | | 1 (1) |  |  |
| *O-Full Thickness Burn* | | 2 (1) | | 2 (4) | | 0 |  |  |
| *O-Physiologic Collapse* | | 1 (1) | | 0 | | 1 (1) |  |  |
| *O-Sequelae of Injury* | | 5 (4) | | 4 (9) | | 1 (1) |  |  |
| *O-Crush* | | 3 (2) | | 2 (4) | | 1 (1) |  |  |
| *O-Other* | | 2 (1) | | 1 (2) | | 1 (1) |  |  |
|  | |  | |  | |  | | |

CTD=catastrophic tissue destruction, CNS=central nervous system, H=haemorrhage, MOF=multi organ failure and sepsis, O=Other.

*#The non-preventable group includes indeterminate cases.*

*^@^The preventable group includes potentially preventable cases.*
